# Multiple CDK inhibitor dinaciclib suppresses neuroblastoma growth via inhibiting CDK2 and CDK9 activity

**DOI:** 10.1038/srep29090

**Published:** 2016-07-05

**Authors:** Zhenghu Chen, Zhenyu Wang, Jonathan C. Pang, Yang Yu, Shayahati Bieerkehazhi, Jiaxiong Lu, Ting Hu, Yanling Zhao, Xin Xu, Hong Zhang, Joanna S. Yi, Shangfeng Liu, Jianhua Yang

**Affiliations:** 1 Department of Ophthalmology, Shanghai Tenth People’s Hospital, Tongji University School of Medicine, Shanghai 200072, P. R. China; 2 Texas Children’s Cancer Center, Department of Pediatrics, Dan L. Duncan Cancer Center, Baylor College of Medicine, Houston, Texas 77030, USA; 3 Department of Pathology, University of Texas MD Anderson Cancer Center, Houston, Texas 77030, USA; 4 Department of Breast Surgery, The second hospital of Jilin University, Changchun, Jilin 130041, China; 5 College of Public Health, Xinjiang Medical University, Urumqi 830011, China; 6 Department of Neurosurgery, Xiangya Hospital, Central South University, Changsha 410008, China; 7 Department of Stomatology, Huashan Hospital, Fudan University, Shanghai 200040, China

## Abstract

Neuroblastoma (NB), the most common extracranial solid tumor of childhood, is responsible for approximately 15% of cancer-related mortality in children. Aberrant activation of cyclin-dependent kinases (CDKs) has been shown to contribute to tumor cell progression in many cancers including NB. Therefore, small molecule inhibitors of CDKs comprise a strategic option in cancer therapy. Here we show that a novel multiple-CDK inhibitor, dinaciclib (SCH727965, MK-7965), exhibits potent anti-proliferative effects on a panel of NB cell lines by blocking the activity of CDK2 and CDK9. Dinaciclib also significantly sensitized NB cell lines to the treatment of chemotherapeutic agents such as doxorubicin (Dox) and etoposide (VP-16). Furthermore, dinaciclib revealed *in vivo* antitumor efficacy in an orthotopic xenograft mouse model of two NB cell lines and blocked tumor development in the TH-MYCN transgenic NB mouse model. Taken together, this study suggests that CDK2 and CDK9 are potential therapeutic targets in NB and that abrogating CDK2 and CDK9 activity by small molecules like dinaciclib is a promising strategy and a treatment option for NB patients.

Neuroblastoma (NB) is the most common extracranial solid tumor in children, accounting for 8–10% of all childhood tumors and 15% of all pediatric cancer related mortality^1^. Although considerable progress of the biological understanding and diagnosis of this deadly malignancy has been made in the past decades, the cure rate has only modestly improved with less than 40% of high-risk NB patients surviving past five years^2^. This poor survival rate (despite one of the most intensive and morbid treatment regimens available) combined with the lack of recurrent, druggable somatic mutations, urgently challenges researchers to identify and drug new targets in NB^3^. To achieve better outcomes in NB, a better understanding of NB biology is critical as the novel therapeutic strategies based on such work would likely benefit patients with high-risk NB.

The cell cycle process is highly conserved in eukaryotes, and the process is strictly controlled to ensure successful cell division. Cyclin-dependent kinases (CDKs) are known for their roles as critical cell cycle regulators when working together with their associated cyclins to promote cell cycle progression^4^,^5^. CDK1, CDK2, CDK4, and CDK6 are involved in DNA replication, mitotic progression, and growth regulatory signals responses, whereas CDK7, CDK8, and CDK9 are important in transcriptional regulation^6^,^7^,^8^,^9^. The Retinoblastoma protein (Rb) has been identified as a tumor suppressor for the role it plays in the negative regulation of the cell cycle^10^. During cell division, Rb binds to the transcription factor E2F and inhibits the activity of the E2F complex, thus preventing cell cycle progression from the G_1_ phase to the S phase^11^,^12^. Phosphorylation of Rb is initiated by the cyclin D/CDK4/CDK6 complex and followed by additional phosphorylation by the cyclin E/CDK2 complex^13^. Rb has been reported to be a CDK2 substrate^14^ and CDK2-mediated Rb phosphorylation at its preferred phosphorylation sites serine 807/811 (Ser807/811) results in the inactivation of Rb and contributes to cell cycle progression^15^,^16^,^17^. CDK9, together with cyclin T1, comprises a positive transcription elongation factor b (P-TEFb), which plays a key role in the regulation of RNA polymerase II (RNAP II)-mediated transcription via phosphorylation of RNAP II at serine 2 (Ser2) in the carboxy-terminal domain^18^. This phosphorylation releases RNAP II from its promoter proximal paused state after transcriptional initiation, triggering transcriptional elongation and ultimately mRNA transcript formation.

Aberrant activation of CDKs results in abnormal cell cycle progression and tumorigenesis^19^. Indeed, small molecule inhibition of CDKs has been reported to have anti-tumor effects in a variety of human cancers, including breast cancer, chronic lymphocytic leukemia (CLL), small lymphocytic lymphoma (SLL), B-cell lymphoma, melanoma, pancreatic cancer, and non-small cell lung cancer (NSCLC)^20^,^21^,^22^,^23^,^24^,^25^. While several of these CDK inhibitors have also demonstrated anti-tumor effects in pre-clinical models of NB or completed clinical trials in NB^26^,^27^,^28^,^29^,^30^,^31^,^32^, the novel CDK inhibitor dinaciclib has not yet been evaluated in patients with NB. Thus, the possible mechanisms of action for dinaciclib in NB have not yet been investigated.

Dinaciclib, a newly developed multiple CDK inhibitor, exerts its cytotoxic effects via inhibiting CDK1, CDK2, CDK5, and CDK9 activity, with a much lower IC50 than that of other CDK inhibitors^33^. Here, we report that by abrogating CDK2 and CDK9 activity, dinaciclib exhibited significant cytotoxicity in all the NB cell lines tested. High expression of CDK2 correlates with poor outcome for NB patients. Dinaciclib induced cell death by blocking the phosphorylation of Rb at Serine 807/811 and of RNAP II at Serine 2 in NB cells. Also, in combination, dinaciclib sensitized NB cells to the treatment of traditional chemotherapeutic drugs like doxorubicin (Dox) and etoposide (VP-16). More importantly, dinaciclib demonstrated *in vivo* anti-tumor efficacy in multiple NB mouse models via inhibiting CDK2 and CDK9 activity. Taken together, our results suggest that CDK2 and CDK9 are potential therapeutic targets in NB and that novel small molecule CDK inhibitors like dinaciclib, alone or in combination with chemotherapeutic agents, should be developed for patients with NB.

## Results

### Multiple CDK inhibitor dinaciclib inhibits NB cell proliferation and induces cell cycle arrest in a panel of NB cell lines

**Figure 1:**
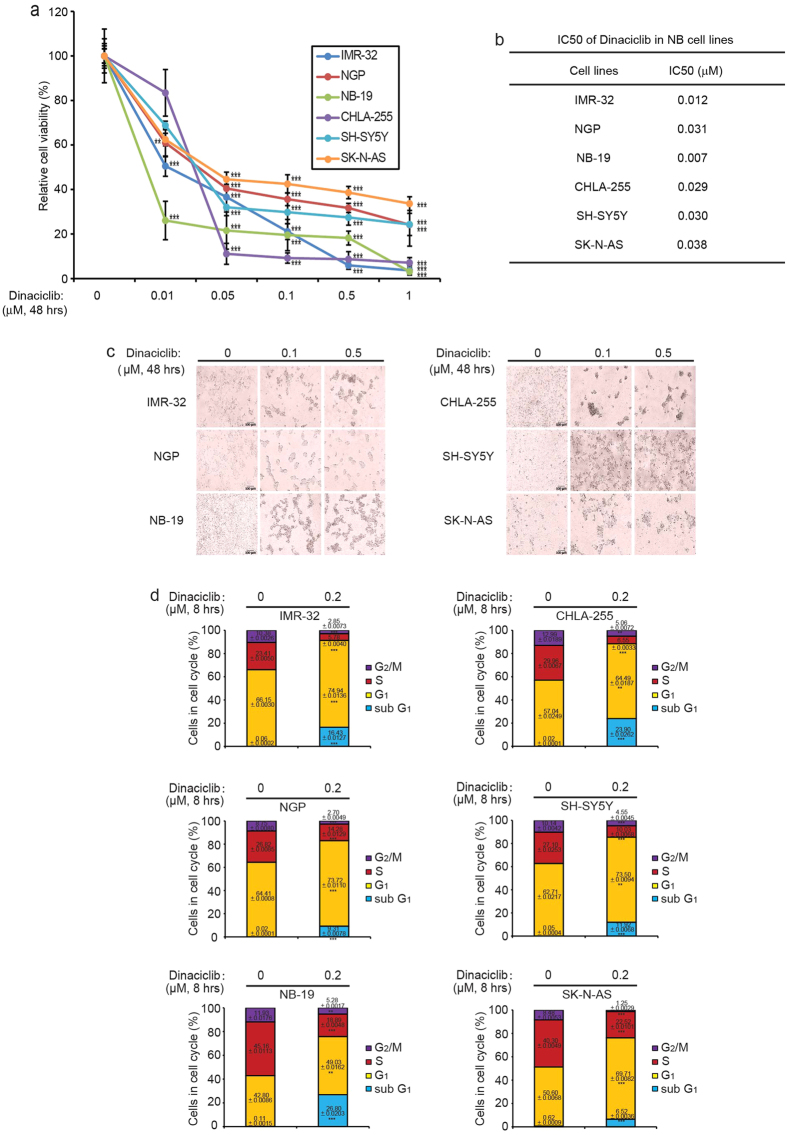
Dinaciclib inhibits NB cell proliferation and induces cell cycle arrest in a panel of NB cell lines. (**a**) Six typical NB cell lines were treated with increasing concentrations of dinaciclib for 48 hrs. Cell viability was then measured by the Cell Counting Kit-8 (CCK-8) assay. *P*-values < 0.01 (**) or *P* < 0.001 (***) (Student’s t-test, two-tailed) were indicated. (**b**) The IC50 values of dinaciclib on each cell line listed were calculated based on the data in (**a**). (**c**) Morphologic changes of six NB cell lines treated with two concentrations of dinaciclib for 48 hrs. (**d**) IMR-32, NGP, and NB-19 cells, as well as CHLA-255, SH-SY5Y and SK-N-AS cells, were treated with 0.2 μM dinaciclib for 8 hrs. Cells were then washed in cold PBS and then fixed in 70% ice cold ethanol, incubated in PI solution and analyzed by flow cytometry. Cell cycle distributions of each cell line were presented as % vehicle ± S.D. *P*-values <0.01 (**), or *P* < 0.001 (***) (Student’s t-test, two-tailed) were indicated.

To explore the cytotoxic effect of dinaciclib in NB cell lines, we chose six typical NB cell lines including IMR-32, NGP, and NB-19, CHLA-255, SH-SY5Y and SK-N-AS cell lines. Treatment with dinaciclib significantly suppressed cell proliferation in a dose-dependent manner in all six of the NB cell lines tested (Fig. 1a). The IC50s of dinaciclib in all the cell lines were relatively low (Fig. 1b). The cell death induced by dinaciclib was further confirmed by cell morphology imaging of the six cell lines after treatment in a dose-dependent manner (Fig. 1c).

To further determine whether the cytotoxicity seen with dinaciclib treatment results from cell cycle arrest, the effect of dinaciclib on cell cycle was assessed in the six NB cell lines. With an increased percentage of cells in the sub G_1_ and G_1_ phases, dinaciclib treatment led to cell cycle arrest in the six NB cell lines tested (Fig. 1d).

### Dinaciclib suppresses colony formation ability of a subset of NB cell lines

**Figure 2:**
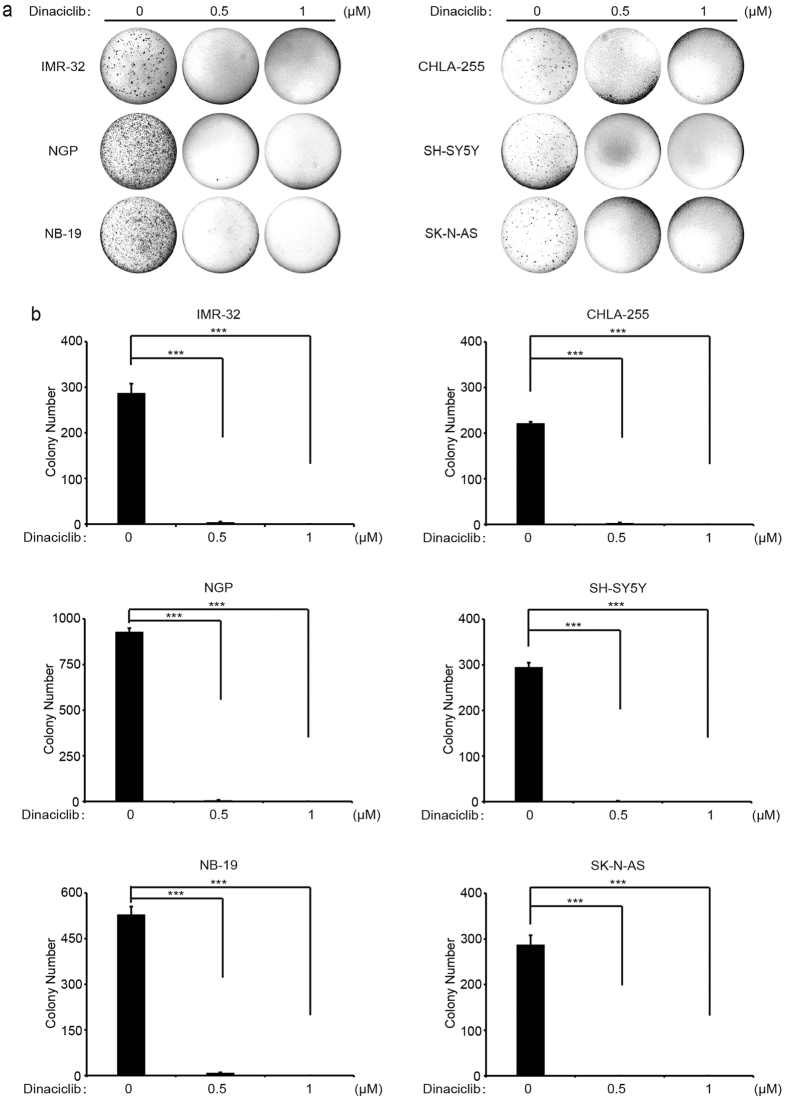
Dinaciclib suppresses colony formation ability of NB cell lines. (**a**) A panel of six NB cell lines were seeded in six-well plates with increasing concentrations of dinaciclib in soft agar. After two to three weeks incubation, crystal violet staining was performed. (**b**) Colony numbers from (**a**) were presented as mean ± S.D. *P*-values < 0.001 (***) (Student’s t-test, two-tailed) were indicated.

The ability to grow in soft agar cultures is one of the distinctive characteristics of tumor cells. To determine whether dinaciclib affects the colony formation ability of NB cell lines, we performed soft agar assays in the six NB cell lines listed above. Consistently, we found that dinaciclib dramatically reduced ability to form colonies in all six NB cell lines tested, compared to the control group (Fig. 2a). Colony numbers were then quantified in each group, which showed that dinaciclib significantly attenuated anchorage-independent NB cell growth (Fig. 2b).

### Dinaciclib abrogates CDK2 and CDK9 activity in NB cells

**Figure 3:**
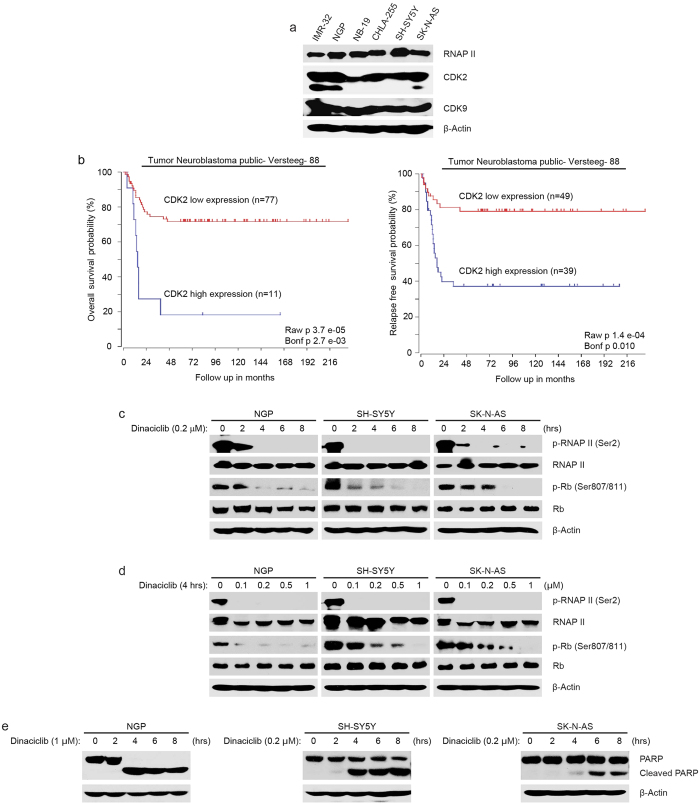
Dinaciclib abrogates CDK2 and CDK9 activity in three NB cell lines. (**a**) Expressions of RNAP II, CDK2 and CDK9 were shown in six NB cell lines. (**b**) Overall survival probability for NB patients with high CDK2 expression (blue; n = 50) and low CDK2 expression (red; n = 38) (Versteeg-88 data set); Relapse-free survival probability for NB patients with high CDK2 expression (blue; n = 39) and low CDK2 expression (red; n = 49) (Versteeg-88 data set). (**c**) NGP, SH-SY5Y and SK-N-AS cells were treated with 0.2 μM dinaciclib for increasing time points, lysed and subjected to immunoblotting with the indicated antibodies. (**d**) NGP, SH-SY5Y, and SK-N-AS cells were exposed to increasing concentrations of dinaciclib for 4 hrs, lysed, and immunoblotted with the indicated antibodies. (**e**) NGP, SH-SY5Y and SK-N-AS cells were treated with the indicated concentrations of dinaciclib for increasing times, and then the cells were collected and lysed, and immunoblotted with anti-PARP antibody. β-Actin antibody was used as a loading control in all samples.

To investigate the molecular mechanisms underlying the sensitivity of NB cells to dinaciclib, we firstly assessed the endogenous expression levels of RNAP II, CDK2, and CDK9 in NB cell lines. As shown in Fig. 3a, all six NB cells showed relatively high expression levels of RNAP II, CDK2, and CDK9, suggesting that CDK2 and CDK9 may be ideal targets in NB therapy. We then examined whether expression of CDK2 and CDK9 have prognostic significance in NB. Kaplan–Meier survival curves were generated based on the data set in R2 gene expression databases (R2: http://r2.amc.nl) and data analysis with the R2 database showed that high expression of the CDK2 gene predicts lower overall and relapse-free survival in the Versteeg-88 data set (Fig. 3b). Consistently, high expressions of both the CDK2 and CDK9 genes predict lower relapse-free survival in the MYCN non-amplified NB patients from the Seeger-102 data set (Supplemental Fig. S1). These findings suggest that CDK2 and CDK9 are potential predictive biomarkers for the prediction of outcomes in NB patients.

As mentioned above, CDK2 directly promotes cell cycle progression in higher eukaryotes by working with its associated cyclins (cyclin A and cyclin E). Inhibition of CDK2 activity results in cell cycle arrest in human breast carcinoma cells^34^ and CDK9 inhibition subsequently causes the inactivation of RNAP II, resulting in cell apoptosis in multiple myeloma cells^35^. Therefore, we hypothesize that the cytotoxicity of dinaciclib in NB cells may similarly result from the inhibition of CDK2 and CDK9 activity.

To test this hypothesis, three NB cell lines (NGP, SH-SY5Y, and SK-N-AS) were treated with dinaciclib at different time points and with various doses. As expected, dinaciclib significantly decreased the phosphorylation levels of Rb at Ser807/811 and of RNAP II at Ser2 in all three cell lines in time- and dose-dependent manners (Fig. 3c,d). Moreover, cell death of NGP, SH-SY5Y and SK-N-AS cells were induced by dinaciclib in a time-dependent manner (Fig. 3e). Taken together, this data demonstrates that dinaciclib induces cytotoxicity in NB cells through the inhibition of CDK2 and CDK9 activity.

### Dinaciclib significantly enhances the cytotoxic effect of Doxorubicin and Etoposide on NB cell lines

**Figure 4:**
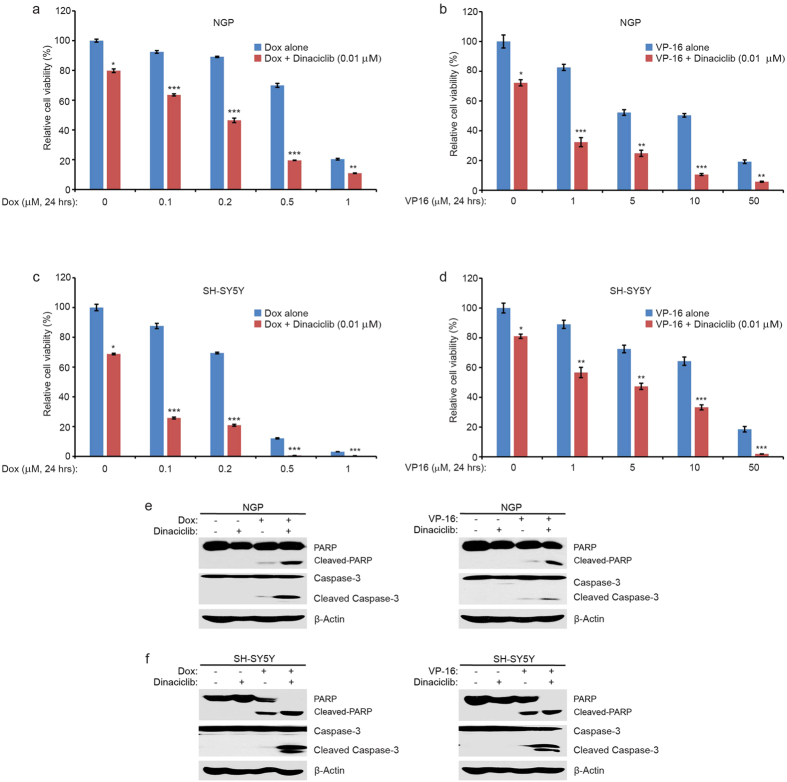
Dinaciclib enhances Dox- and VP-16-induced cytotoxicity in NGP and SH-SY5Y cell lines. (**a**,**c**) NGP and SH-SY5Y cells were seeded in 96-well plates and were incubated with the indicated concentrations of Dox plus DMSO or dinaciclib (0.01 μM) for 24 hrs. Cell viability assessed by the CCK-8 assay as described in Methods. (**b**,**d**) NGP and SH-SY5Y cells were similarly treated with VP-16 plus DMSO or dinaciclib (0.01 μM) for 24 hrs. Results are represented as % vehicle ± SD. *P*-values < 0.05 (*), *P* < 0.01 (**), or *P* < 0.001 (***) (Student’s t-test, two-tailed) were indicated. (**e**) NGP cells were treated with either Dox (2 μM) or VP-16 (5 μM) or dinaciclib (0.05 μM) alone or the combination of dinaciclib (0.05 μM) with Dox (2 μM) or VP-16 (5 μM) for 8 hrs. (**f**) SH-SY5Y cells were treated with either Dox (1 μM) or VP-16 (5 μM) or dinaciclib (0.05 μM) alone or the combination of dinaciclib (0.05 μM) with Dox (1 μM) or VP-16 (5 μM). All samples were collected and subjected to SDS-PAGE, and immunoblotted with anti-PARP or Caspase-3 antibody. β-Actin antibody was used as a loading control for whole cell extracts in all samples.

Given the increased cell cycling of tumor cells, we reasoned that the pharmacological inhibition of CDK2 and CDK9 would increase the sensitivity of NB cells to chemotherapeutic agents. To test this hypothesis, NGP and SH-SY5Y cells were treated with doxorubicin (Dox) or etoposide (VP-16) alone or in combination with dinaciclib at a relatively low dose. As shown in Fig. 4a,b, we found that the cytotoxic effect of Dox or VP-16 on NGP cells were significantly enhanced when treated in combination with low doses of dinaciclib. Similar results were observed when SH-SY5Y cells were treated with Dox or VP-16 in combination with dinaciclib (Fig. 4c,d). Consistently, dinaciclib enhanced Dox- and VP-16-induced cell death in NGP cells, as the expression levels of ADP-ribose polymerase (PARP) and Caspase-3 cleavage increased in the combination treatment group compared to the dinaciclib, Dox, or VP-16 single treatment groups (Fig. 4e). Dinaciclib also sensitized SH-SY5Y cells to Dox and VP-16 induced cell death by working together with Dox and VP-16 (Fig. 4f). Together, these results demonstrate that dinaciclib enhances the cytotoxic effects of Dox and VP-16 on NB cell lines by increasing Dox- and VP-16-induced cell death.

### Dinaciclib strongly inhibits NB tumor growth by blocking CDK2 and CDK9 activity in an orthotopic NB xenograft mouse model

An orthotopic NB xenograft mouse model was utilized to test the *in vivo* efficacy of dinaciclib. Luciferase gene-transduced NGP and SH-SY5Y cells were surgically injected into the left renal capsule of nude mice. Two weeks after injection, tumor signals were captured and analyzed by bioluminescent imaging. Standardized by a threshold of 1 × 10^7^ total flux (p/s), tumor-bearing mice with each injected cell line were randomly divided into two groups, and the mice were then treated with either dinaciclib or an equal volume of dimethyl sulfoxide (DMSO) (carrier control). Dinaciclib was administered alone by intraperitoneal injection at 20 mg/kg daily for 21 days.

**Figure 5:**
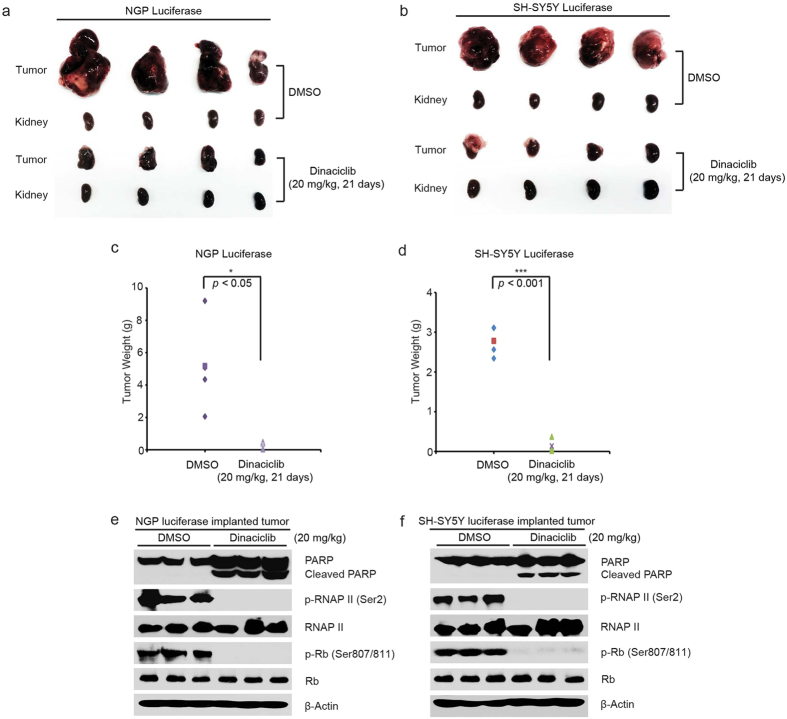
Dinaciclib inhibits tumor growth in an orthotopic NB xenograft mouse model. (**a**,**b**) At the end of treatment, photos of NGP and SH-SY5Y xenografted tumors and the control kidney from DMSO control group and and dinaciclib treated group (20 mg/kg) were shown. (**c**,**d**) NGP- and SH-SY5Y-derived tumor weights from control (N = 4) and treatment groups (N = 4) were presented. *P*-values < 0.05 (*) and *P* < 0.001 (***) (Student’s t-test, two-tailed) were indicated. (**e**,**f**) The mice bearing NGP and SH-SY5Y xenografted tumors for five weeks were treated with 20 mg/kg of dinaciclib by intraperitoneal injection once daily for two days. The mice were then sacrificed, and the tumors were harvested and lysed for immunoblotting with the indicated antibodies. β-Actin was used as a loading control.

Significant tumor regression in the dinaciclib-treated group was observed upon comparison with the control group in the NGP mouse tumor model (Fig. 5a,c). In addition, similar results were observed in the orthotopic NB mouse model using SH-SY5Y cells with luciferase gene expression (Fig. 5b,d). To assess the effect of dinaciclib on CDK2 and CDK9 activity *in vivo*, we treated the tumors bearing mice with dinaciclib at 20 mg/kg daily for two days and harvested the tumors for analysis of CDK2 and CDK9 activities. As expected, dinaciclib strongly blocked the phosphorylation of Rb (Ser807/811) and RNAP II (Ser2) and induced the cleavage of PARP in those tumors (Fig. 5e,f). Collectively, these results showed that the inhibition of CDK2 and CDK9 activity by dinaciclib could block NB growth and induce tumor cell death *in vivo*.

### Dinaciclib blocks NB tumor development in the TH-MYCN transgenic mouse model

**Figure 6:**
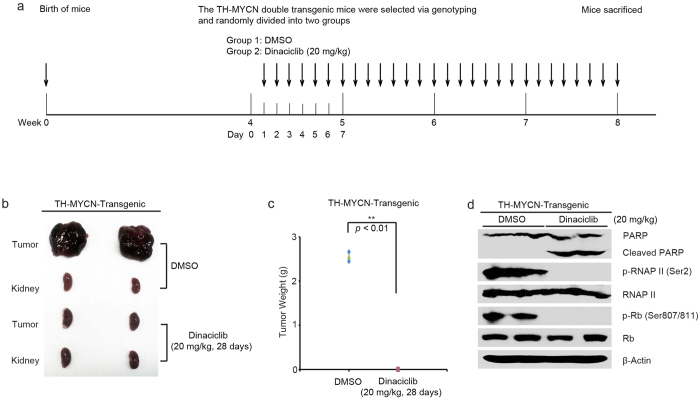
Dinaciclib blocks tumor development in the TH-MYCN NB transgenic mouse model. (**a**) Treatment strategy used in this study. (**b**) Pictures of tumors and the corresponding kidney in each group (N = 2) at the end of the study. (**c**) Tumor weights from the last day of treatment. Data was collected with two mice per group. *P*-value < 0.01 (**) (Student’s t test, two-tailed) was indicated. (**d**) The seven-week-old TH-MYCN NB transgenic mice were treated with 20 mg/kg of dinaciclib by intraperitoneal injection once daily for two days. The mice were then sacrificed, and the tumor tissues were harvested and lysed for immunoblotting with the indicated antibodies. β-Actin was used as a loading control.

MYCN has long been considered as one of the major oncogenic drivers in the progression of NB, and patients with MYCN amplified status are considered “high risk”^36^. We assessed the efficacy of dinaciclib in the TH-MYCN transgenic mouse model (the most widely used murine NB model)^37^,^38^,^39^. The homozygous TH-MYCN transgenic mice at four weeks of age were divided randomly into two groups. The mice were treated by 20 mg/kg dinaciclib or an equal volume of DMSO daily by intraperitoneal injection for four weeks (Fig. 6a). We found that dinaciclib blocked tumor development in TH-MYCN transgenic mice when compared with the control group (Fig. 6b,c).

Another group of homozygous TH-MYCN transgenic mice at seven weeks of age were treated with either dinaciclib (20 mg/kg) or an equal volume of DMSO once daily for two days. Consistently, dinaciclib blocked CDK2 and CDK9 activity by abolishing the phosphorylation of Rb (Ser807/811) and RNAP II (Ser2) and ultimately induced PARP cleavage in those tumor tissues (Fig. 5d). Together, these results showed that the inhibition of CDK2 and CDK9 activity by dinaciclib could block NB tumor development and induce tumor cell death in TH-MYCN transgenic mouse model.

## Discussion

Dysregulation of the cell cycle has been considered as a hallmark of almost all human cancers and is always associated with abnormal activation of CDKs^4^,^40^. Therefore, CDKs are potential targets in NB, and pharmacological inhibition of important CDKs is a viable strategy to treat NB patients. In this study, we found that dinaciclib significantly inhibited both anchorage-dependent and independent growth in a panel of neuroblastoma cells by abrogating CDK2 and CDK9 activity. Furthermore, dinaciclib sensitized two typical NB cell lines, NGP and SH-SY5Y, to apoptosis induced by two traditional chemotherapeutic agents Dox and VP-16. More importantly, dinaciclib significantly inhibited NB tumor growth in two orthotopic mouse models by blocking CDK2 and CDK9 activity and inducing tumor cell death. Dinaciclib also blocked tumor development in the TH-MYCN transgenic NB mouse model.

CDKs, which constitute a large family of protein kinases that play various roles in eukaryotic cells and cell cycle control, are an attractive target for anti-cancer therapy. Therefore, developing effective, selective, and potent CDK inhibitors as novel therapeutic agents, has been a hotspot in cancer therapy. Particularly, CDK2 and CDK9 are reported to be potential targets for drug development for cancer therapy^41^,^42^. The first generation of multiple CDK inhibitors, flavopiridol, induces cell cycle arrest in dividing cells^25^. The spectrum of flavopiridol’s ATP-competitive inhibition activity includes CDK1, CDK4, and CDK9 34,43,44. Flavopiridol is also capable of inducing apoptosis and promoting cell death independent of p53^45^. However, flavopiridol treatment decreases mitochondrial oxygen consumption and autophagy, which protects cells against flavopiridol-mediated apoptosis^46^. Another limitation of flavopiridol is that (in CLL cells) it causes endoplasmic reticulum (ER) stress leading to flavopiridol resistance, which may be the mechanism underlying relapse in patients treated with flavopiridol^47^. Less information is known about other CDK inhibitors (e.g. BAY-1000394, P276-00, LEE011, R547, etc). As they are still under investigation, and few of them have undergone scrutiny in human trials^48^.

As a second generation multiple CDK inhibitor, dinaciclib (SCH-727965, MK-7965) has a much broader therapeutic index than flavopiridol^49^. Moreover, dinaciclib is more effective and selective on its targeted CDKs compared with flavopiridol^33^. Another advantage of dinaciclib is that it induces apoptosis in CLL cells irrespective of the presence of traditionally negative prognostic factors like unmutated Ig heavy chain V-III region VH26 (IGHV), prior fludarabine exposure, or del (17p13.1) status^50^. Despite the fact that dinaciclib as a single agent has been approved to enter phase II clinical trials for CLL and phase III clinical trials in patients with refractory CLL and SLL, the survival rate and relapse rate of the patients after dinaciclib treatment remains to be determined^41^. Its efficacy in NB and other diseases needs to be explored as well. It has been reported that the most common side effect dinaciclib caused in clinical trials was low white blood cell counts. Other side effects include diarrhea, fatigue, and blurred vision^51^. These side effects should be taken into consideration before dinaciclib is applied to NB patients.

Curing NB via small molecule CDK inhibition has been an active field in cancer research. CDK7 inhibitor THZ1 has been shown to suppress global MYCN-dependent transcription in NB, but it is less potent in MYCN non-amplified NB cell lines^26^. Dual inhibition of CDK4/CDK6 by LEE011 is reported to cause cell cycle arrest and induces senescence in 12 out of 17 neuroblastoma cell lines, but not all the tested cell lines were sensitive to LEE011^27^. These studies shed light on the application of CDK inhibitors in the treatment of NB and on the urgent need to develop novel and potent CDK inhibitors to achieve better outcomes in NB. Other CDK inhibitors that target a single CDK, like AT7519^28^, or multiple CDKs, like Lupiwighteone^29^, or a combination of several CDK inhibitors like roscovitine and CR8^31^, are alternate options but the efficacy of these drugs in NB is largely unknown. On the other hand, as demonstrated in our data, dinaciclib remarkably decreases the cell viability of all NB cell lines tested, which indicates that it is highly effective *in vitro*. Furthermore, it also exerts significant *in vivo* anti-tumor effects in both the orthotopic NB xenograft mouse model and the TH-MYCN transgenic NB mouse model. Dinaciclib has also been reported to inhibit unfolded protein response in a CDK1- and 5-dependent manner^52^. Since dinaciclib targets four CDKs and only two of them were investigated in this paper, it is highly likely that it also inhibits CDK1 and/or CDK5 activity in NB as well. Data analysis in the R2 database demonstrates that high expressions of both CDK2 and CDK9 genes correlate with the poor outcomes of NB patients in the Versteeg-88 data set (Supplemental Figs S2–S3). To explore the possible anti-tumor effects of CDK1/5 inhibition caused by dinaciclib, NB-19 and SK-N-AS cell lines were chosen to perform the cell viability assay. The CDK1/5 inhibitor alone had limited cytotoxic effect on the two NB cell lines tested, whereas the CDK2/9 inhibitor alone significantly inhibited NB cell proliferation (Supplemental Fig. S4). Interestingly, the CDK1/5 inhibitor and the CDK2/9 inhibitor showed synergistic effects on the inhibition of NB cell proliferation (Supplemental Fig. S5). Compared with the CDK1/5 inhibitor alone, the CDK2/9 inhibitor alone, or their combinations, dinaciclib exerted a stronger cytotoxic effect with a much lower dose, due to the low IC50s of dinaciclib on its targeted CDKs (Supplemental Fig. S5). These data suggest that the inhibition of CDK2/9 plays a predominant role whereas the inhibition of CDK1/5 has an auxiliary role in dinaciclib induced toxicity in NB.

Chemoresistance has frequently been considered as one of the major causes of relapse in patients, especially in high-risk NB patients. Therefore, clarifying the molecular mechanisms that are responsible for chemoresistance is of vital importance. Since dinaciclib shows cytotoxicity in NB cell lines by blocking the activity of its targeted CDKs, we reasoned that dinaciclib might augment the sensitivity of NB cell lines to Dox and VP-16 treatment. This study demonstrates that dinaciclib indeed significantly enhanced the sensitivity of two typical NB cell lines, NGP and SH-SY5Y to Dox and VP-16 treatment by blocking CDK2 and CDK9 activity. Furthermore, the combination of Dox or VP-16 with dinaciclib dramatically induced cell death compared with Dox or VP-16 treatment alone at the indicated time points. As SH-SY5Y cells are less resistant to chemotherapy than NGP cells, we found that dinaciclib is more potent in sensitizing SH-SY5Y cells to Dox and VP-16 induced cell death. These results suggest that the combination of the multiple CDKs inhibitor dinaciclib with traditional chemotherapeutic drugs might be an effective strategy for treating NB patients. Dinaciclib was developed through a mass scale screening strategy, which included diverse assays with parameters such as pharmacokinetics, safety characteristics, etc^49^. This functional analysis based strategy is highly effective and will allow rapid screening for novel anti-tumor compounds like dinaciclib in the future.

In conclusion, the small molecule dinaciclib inhibits the activity of CDK2 and CDK9 in NB cell lines, resulting in the inhibition of cell proliferation and the induction of cell death both *in vitro* and *in vivo*. Promising combinatorial effects are also seen with traditional chemotherapeutic agents. Our data suggests that CDKs, particularly CDK2 and CDK9, are promising therapeutic targets in NB and our study offers the possibility for the rational use of CDK inhibitors like dinaciclib as a new approach for the treatment of NB patients.

## Materials and Methods

### Antibodies and Reagents

Multiple CDK inhibitor dinaciclib was purchased from Selleckchem (S2768) (Selleckchem, Houston, TX, USA). Anti-β-Actin (A2228) antibodies, Doxorubicin (Dox, D1515), and etoposide (VP-16, E1383) were from Sigma (Sigma-Aldrich Corp, St. Louis, MO, USA). Anti-phospho-RNAP II (Ser2) (A300-654A) and anti-RNAP II (A300-653A) antibodies were from Bethyl Laboratories (Bethyl Laboratories, Inc., Montgomery, TX, USA). Anti-CDK2 (M2) (sc-163), anti-CDK9 antibodies (D-7) (sc-13130), CDK1/5 inhibitor (sc-202094), and CDK2/9 inhibitor (sc-221411) were from Santa Cruz Biotechnology (Santa Cruz Biotechnology, Dallas, TX, USA). Anti-PARP (9532 S), anti-Caspase-3 (9662S), anti-phopho-Rb (Ser807/811) (9308S), and anti-Rb (9309S) primary antibodies, as well as anti-Mouse (7076S) and anti-Rabbit (7074S) secondary antibodies were from Cell Signaling Technology (Cell Signaling Technology, Danvers, MA, USA).

### Cell Lines and Cell Culture

The MYCN-non-amplified (CHLA-255, SH-SY5Y and SK-N-AS) and the MYCN-amplified (IMR-32, NGP and NB-19) human NB cell lines used in this study were cultured in RPMI Medium 1640 (RPMI) (Lonza, Walkersville, MD, USA) complemented with 10% (v/v) heat-inactivated Fetal Bovine Serum (FBS) (SAFC Biosciences, Lenexa, KS, USA), 100 μg/mL streptomycin, and 100 units/mL penicillin. All cells were maintained in a humidified incubator with 5% CO_2_ at 37 °C. All experiments listed in this study were performed with cells under exponential growth conditions. The NB-19 cell line was from Dr. A. Davidoff (St. Jude Children’s Research Hospital).

### Cell Viability Assay

Cell Counting Kit-8 (CCK-8, WST-8[2-(2-methoxy-4-nitrophenyl)-3-(4-nitrophenyl)-5-(2,4-disulfophenyl)-2 H-tetrazolium, monosodium salt]) (Dojindo Laboratories, Rockville, MA, USA) was used to conduct the assays. Cells were seeded and kept in 96-well clear-bottom plates starting from 1 × 10^4^ cells per well. The media were changed 24 hrs later, and various concentrations of dinaciclib, Dox, VP-16, or their combinations were added to the plates. Cells were then maintained at 37 °C for either 24 hrs or 48 hrs. Additionally, NB-19 or SK-N-AS cells were seeded and kept in 96-well plates at 1 × 10^4^ cells per well. After changing the media 24 hrs later, the cells were treated with increasing doses of dinaciclib, CDK1/5 inhibitor, CDK2/9 inhibitor, or their combinations for either 36 hrs or 48 hrs. A mixture of 190 μL of RPMI with 10% FBS and 10 μL of CCK-8 was then added into each well; one hour later, using a microplate reader, the absorbance was measured at 450 nm. Background reading of the media was subtracted from each well, and each experiment was performed in six replicates.

### Cell Imaging

All the NB cell lines used in the study were cultured in 96-well plates starting from 1 × 10^4^ cells per well. After either 24 hrs or 48 hrs of treatment with the indicated concentrations of dinaciclib, cell morphologies were photographed using an optical microscope. Each result was performed in triplicate, and the representative was shown.

### Cell Cycle Analysis

All the tested NB cells were cultured as described above and treated with dinaciclib (0.2 μM) for 8 hrs. At the end of treatment, the cells were washed with ice cold PBS twice and centrifuged for 5 min at 500 g. The resulting pellets were resuspended in 1 ml ice cold PBS, followed by 4 ml 70% ice cold ethanol. The fixed cells were then kept at 4 °C overnight. Propidium iodide (PI) solution, which consisted of 50 μg/mL PI (Biotium) and 100 μg/mL RNaseA, was made and then 1 mL of solution was added to the cell pellets for one hour at 37 °C. Then the stained cells were filtered through a 40 μm mesh. After that, flow cytometry was used within one hour to analyze the samples. By using FCS Express 4 software, the data was analyzed to demonstrate the distribution of the four cell cycle phases (sub G_1_, G_1_, S, G_2_/M). Unstained cells were used as negative control in the analysis.

### Colony Formation Assay

The colony formation assay was performed as previously described^53^,^54^. Briefly, agar (214220, Difco Laboratories, Detroit, MI, USA) was added into distilled water, and the mixture was then autoclaved for 50 min before cooling it down in a 56 °C water bath to make the 5% (w/v) agar. This solution was then mixed with RPMI supplemented with 10% FBS to make bottom agar layer.

For the bottom agar layer, 2 mL of the 0.5% agar/RPMI solution were added to each well and the solution was cooled down to semi-solid. The top agar layer, 1.5 ml of mixture including 0.3% agar and the six NB cell lines at 1 × 10^4^ cells per well, were added on the top of bottom agar. Twenty four hours later, the cells in the culture were treated with the indicated concentrations of dinaciclib. Cells were maintained at 37 °C for 2 to 3 weeks before staining with 500 μL of 0.005% crystal violet (C3886, Sigma) for 4 hrs. Images were captured by the microscope, and colonies were counted 4 hrs later.

### Immunoblotting

The experiments were performed as described previously^55^. Briefly, at the end of each treatment, cells were washed with ice cold PBS twice and lysed at 4 °C for 30 min in cooled RIPA buffer (50 mM Tris-HCl at pH 7.4, 150 mM NaCl, 1 mM EDTA, 1% NP-40, 0.25% sodium deoxycholate, 1 mM phenylmethylsulfonyl fluoride, 1 mM benzamidine, 10 μg/mL leupeptin, 1 mM dithiothreitol, 50 mM sodium fluoride, 0.1 mM sodium orthovanadate, and phosphatase inhibitor cocktail 2 and 3 (p5726 and p0044, Sigma)). The supernatants were collected after centrifuging at 13,000 rpm for 15 min. Bradford reagent (Bio-Rad Laboratories, Hercules, CA, USA) was used to detect protein concentrations, and each sample was mixed with 4× loading buffer and heated at 100 °C for 7~10 min. The proteins were then separated by SDS-PAGE gels, transferred to polyvinylidence fluoride (PVDF) membranes (Bio-Rad), blocked with 5% milk for one hour at room temperature (25 °C), and probed with the suggested dilutions of indicated primary antibodies at 4 °C overnight. The membranes were then incubated with anti-mouse or rabbit secondary antibody conjugated with horseradish peroxidase at room temperature for one hour. Chemiluminescent visualization was conducted by using the ECL-Plus Western detection system (GE Health Care, Buckinghamshire, UK). β-Actin was used as a loading control for whole cell extracts in all groups tested.

### Antitumor Efficacy in Orthotopic Mouse Models of NB

All the athymic NCR nude mice used in this study were purchased from Taconic (Taconic, Hudson, NY, USA) and kept under barrier conditions (pathogen-free conditions provided by plastic cages with sealed air filters). The establishment of the orthotopic mouse model of NB was conducted by implanting NB cells intrarenally as described previously^56^. Briefly, a transverse incision was generated over the left flank of the nude mice, and 1.5 × 10^6^ human luciferase-transduced NGP or SH-SY5Y cells, which were diluted in 0.1 ml of PBS, were surgically injected into the left renal capsule and toward the superior pole of the left kidney of the nude mice.

After allowing the injected mice to engraft for 2 to 3 weeks, tumor-bearing mice with similar sizes (using bioluminescent imaging to monitor the tumor size) were randomly divided into two groups: a DMSO control group and a dinaciclib treated group (20 mg/kg by intraperitoneal (i.p.) injection once daily for 21 days). Each group contained four mice with NGP-luciferase tumors or four mice with SH-SY5Y-luciferase tumors. At the end of the treatment, all mice were sacrificed. Tumors and the right kidneys (control) were harvested, weighed and photographed.

To perform the protein immunoblotting, two groups of NGP- or SH-SY5Y-implanted NB orthotopic mice with similar sizes were treated with either DMSO or dinaciclib (20 mg/kg by i.p. injection) daily for two days. Two days later, the mice were sacrificed, the tumors were harvested, and the samples were lysed for immunoblotting. All mice were handled according to protocols approved by the Institutional Animal Care and Use Committee of the Baylor College of Medicine.

### Antitumor Efficacy in the TH-MYCN transgenic Mouse Model of NB

Homozygous TH-MYCN transgenic mice were obtained via PCR genotyping. At four weeks of age, they were divided into two groups randomly. 20 mg/kg dinaciclib or an equal volume of DMSO was administered daily by i.p. injection to the mice for 28 days. All the mice were sacrificed at the end of the treatment, and the tumors of each group and the corresponding kidneys were photographed and weighed.

To examine the inhibitory effect of dinaciclib on the tumor of TH-MYCN transgenic mice, seven-week-old homozygous TH-MYCN transgenic mice were treated with 20 mg/kg dinaciclib or an equal volume of DMSO once daily for two days. At the end of treatment, the mice were sacrificed and the tumors were collected. The tumor tissues were then lysed for protein immunoblotting with the indicated antibodies. All mice were handled according to protocols approved by the Institutional Animal Care and Use Committee of the Baylor College of Medicine.

### Statistical Analysis

To determine the statistical significance of all the *in vitro* and *in vivo* assays, a two-tailed Student’s t-test was used. All values were presented as mean ± standard deviation (SD). Each assay had been repeated at least twice and the representative data was presented. *P* < 0.05 was considered to be statistically significant in all assays.

## Additional Information

**How to cite this article**: Chen, Z. *et al*. Multiple CDK inhibitor dinaciclib suppresses neuroblastoma growth via inhibiting CDK2 and CDK9 activity. *Sci. Rep.* **6**, 29090; doi: 10.1038/srep29090 (2016).

## Supplementary Material

Supplementary Information
