# Supplementary material for: Multiple CDK inhibitor dinaciclib suppresses neuroblastoma growth via inhibiting CDK2 and CDK9 activity
Source: Sci Rep. 2016 Jul 5;6:29090. doi: 10.1038/srep29090 (PMC4932496; doi:10.1038/srep29090)
Supplement: Supplementary Information [file srep29090-s1.pdf]

Supplemental information

Multiple CDK inhibitor Dinaciclib suppresses neuroblastoma growth via inhibiting CDK2 and CDK9 activity

Zhenghu Chen[1,2], Zhenyu Wang[3,4], Jonathan C. Pang[2], Yang Yu[2], Shayahati Bieerkehazhi[3,5], Jiaxiong Lu[1,2], Ting Hu[2,6], Yanling Zhao[2], Xin Xu[2], Hong Zhang[3], Joanna S. Yi[2], Shangfeng Liu[1,7]*, and Jianhua Yang[2]*

[1]Department of Ophthalmology, Shanghai Tenth People's Hospital, Tongji University School of Medicine, Shanghai 200072, P. R. China

[2]Texas Children's Cancer Center, Department of Pediatrics, Dan L. Duncan Cancer Center, Baylor College of Medicine, Houston, Texas 77030, USA

[3]Department of Pathology, University of Texas MD Anderson Cancer Center, Houston, Texas 77030, USA

[4]Department of Breast Surgery, The second hospital of Jilin University, Changchun, Jilin 130041, China

[5]College of Public Health, Xinjiang Medical University, Urumqi 830054, China

[6]Department of Neurosurgery, Xiangya Hospital, Central South University, Changsha 410008, China

[7]Department of Stomatology, Huashan Hospital, Fudan University, Shanghai 200040, China

*Correspondence to:

Shangfeng Liu, Department of Stomatology, Huashan Hospital, Fudan University, Shanghai 200040, China. Tel/fax: +86-21-6562662. Email: shangfengliufudan@163.com. Jianhua Yang, Texas Children's Cancer Center, Department of Pediatrics, Dan L. Duncan Cancer Center, Baylor College of Medicine, Houston, TX 77030. USA. Phone: 1-832-824-4572, Fax: 1-832-825-1206. Email: jianhuay@bcm.edu;

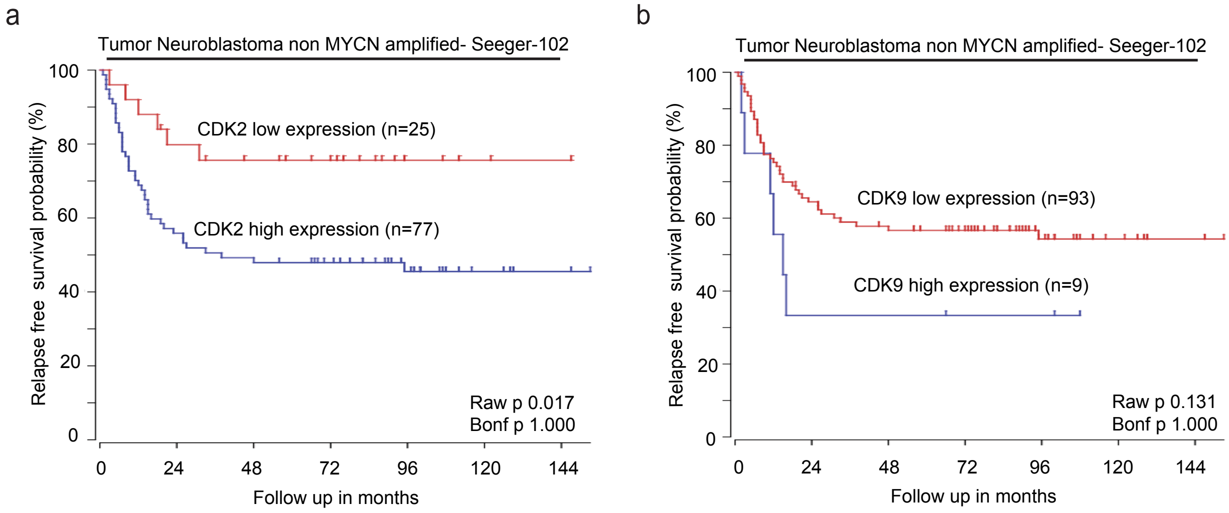

Supplemental Figure S1. Correlations of the expressions of CDK2 and CDK9 with the outcomes in NB patients. (**a**) Relapse-free survival probability for NB patients with high CDK2 expression (blue; n=77) and low CDK2 expression (red; n=25) (Seeger-102 data set). (**b**) Relapse-free survival probability for NB patients with high CDK9 expression (blue; n=9) and low CDK9 expression (red; n=93) (Seeger-102 data set).

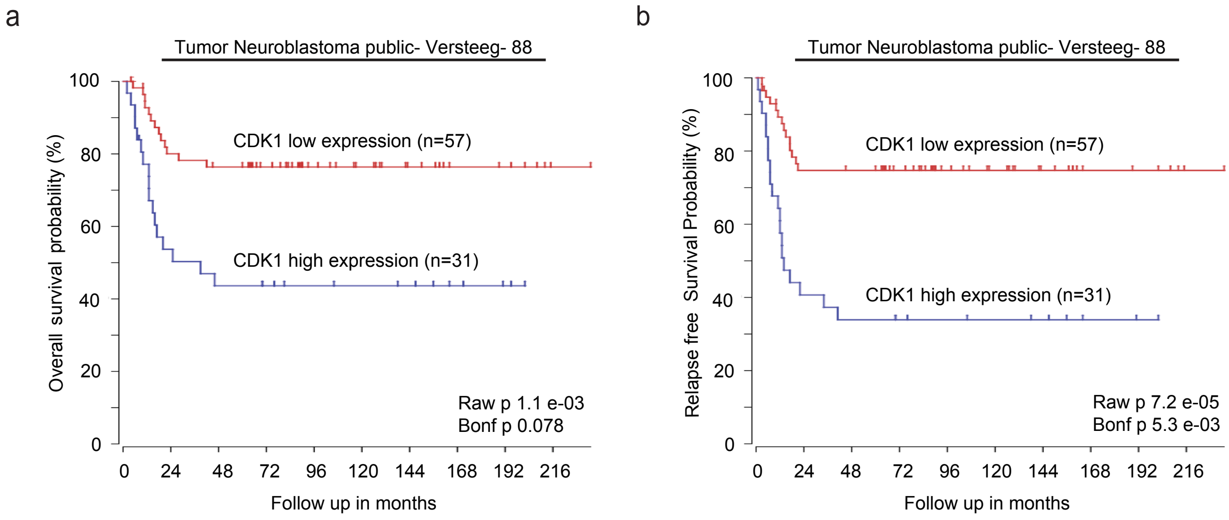

Supplemental Figure S2. Correlations of the expressions of CDK1 with the outcomes in NB patients. (**a**) Overall survival probability for NB patients with high CDK1 expression (blue; n=31) and low CDK1 expression (red; n=57) (Versteeg-88 data set). (**b**) Relapse-free survival probability for NB patients with high CDK1 expression (blue; n=31) and low CDK1 expression (red; n=57) (Versteeg-88 data set).

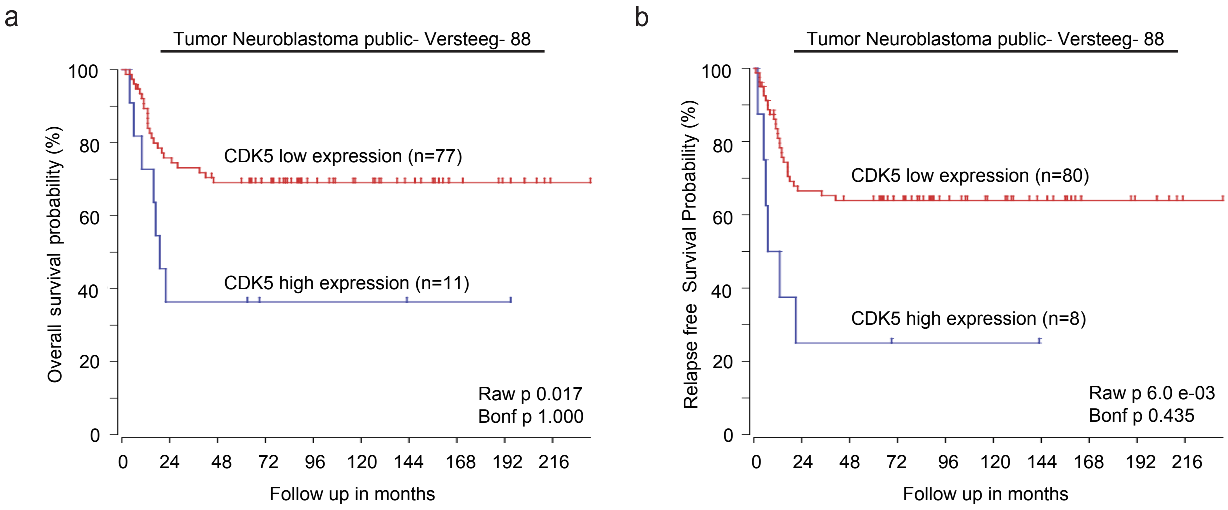

Supplemental Figure S3. Correlations of the expressions of CDK5 with the outcomes in NB patients. (**a**) Overall survival probability for NB patients with high CDK5 expression (blue; n=11) and low CDK5 expression (red; n=77) (Versteeg-88 data set). (**b**) Relapse-free survival probability for NB patients with high CDK5 expression (blue; n=8) and low CDK5 expression (red; n=80) (Versteeg-88 data set).

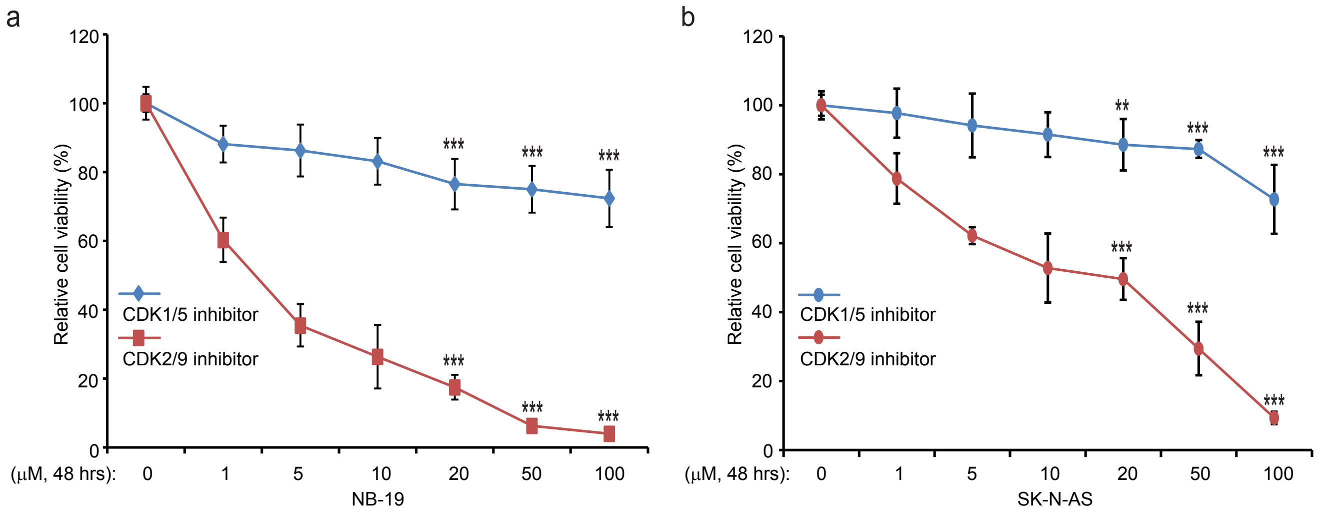

Supplemental Figure S4. The cytotoxic effects of CDK1/5 inhibitor and CDK2/9 inhibitor on NB-19 and SK-N-AS cells. NB-19 (**a**) and SK-N-AS (**b**) cells were treated with increasing concentrations of either CDK1/5 dual inhibitor or CDK2/9 dual inhibitor for 48 hrs. Cell viability was then measured by the Cell Counting Kit-8 (CCK-8) assay. P-values <0.01 (**) or P <0.001 (***) (Student's t-test, two-tailed) were indicated.

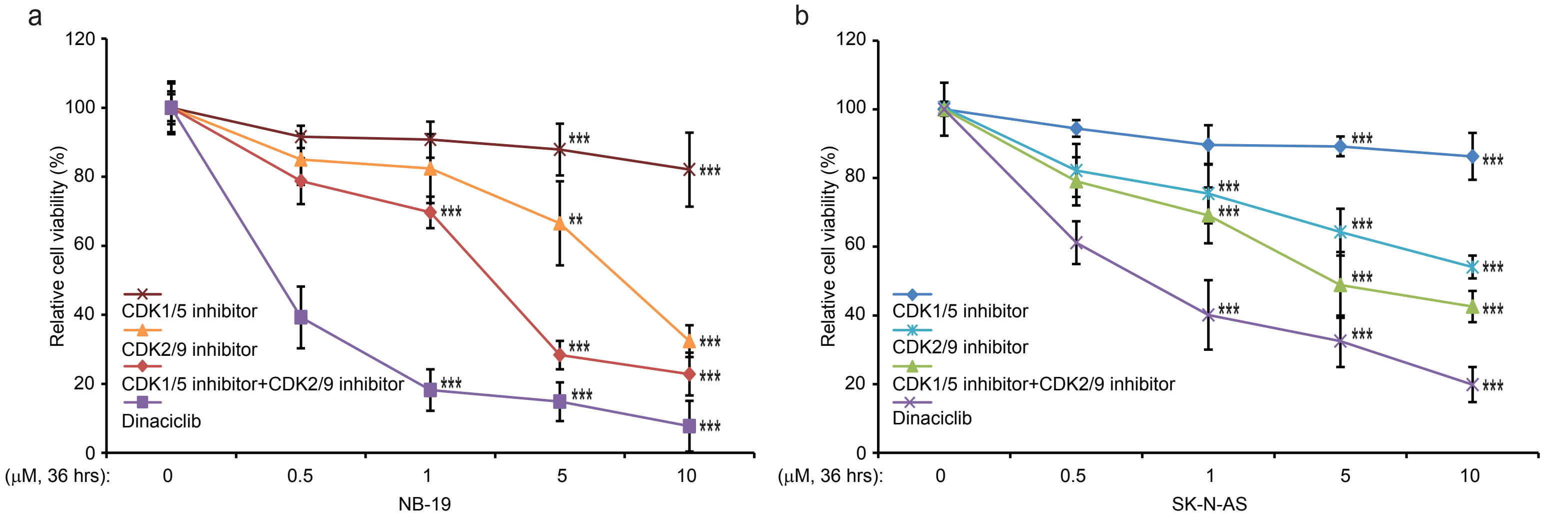

Supplemental Figure S5. The comparison of the cytotoxic effects caused by CDK inhibitors on NB-19 and SK-N-AS cells. NB-19 (**a**) and SK-N-AS (**b**) cells were treated with increasing concentrations of CDK1/5 dual inhibitor, or CDK2/9 dual inhibitor, or their combinations, or Dinaciclib for 36 hrs. Cell viability was then measured by the Cell Counting Kit-8 (CCK-8) assay. P-values <0.001 (***) (Student's t-test, two-tailed) were indicated.
